# Time to Onset of Dysphagia Following Head and Neck Radiation

**DOI:** 10.1007/s00455-024-10782-3

**Published:** 2024-11-14

**Authors:** E. Marin Miller, Rameen K. Walters, Shaun A. Nguyen, Jennifer L. Harper, Bradley Depaoli, Ashli K. O’Rourke

**Affiliations:** 1 Langone Health, Department of Otolaryngology – Head and Neck Surgery, New York University, New York, NY USA; 2 Department of Otolaryngology, Head and Neck Surgery, Temple University, Philadelphia, PA USA; 3 Department of Otolaryngology - Head and Neck Surgery, Medical University of South Carolina, Charleston, SC USA; 4 Department of Radiation Oncology, Medical University of South Carolina, Charleston, SC USA

**Keywords:** Dysphagia, Radiation, Head and neck cancer

## Abstract

To evaluate the time of onset of dysphagia in a cohort of head and neck cancer patients treated with radiation or chemoradiation. Retrospective chart review of adult patients. 237 patients met inclusion criteria for the study. The average age at cancer diagnosis was 62 years (± 12.6) in a predominantly male cohort (*n* = 198, 83.5%). The most common subsite was oropharyngeal (*n* = 146, 60.8%) and squamous cell carcinoma in origin (*n* = 232, 97.9%). Of head and neck cancer patients diagnosed with new onset dysphagia or a dysphagia related diagnosis, nine (3.8%) were diagnosed at six months to 1 year, 12 (5.1%) at 1–2 years, and 17 (7.1%) at greater than 2 years. The mean radiation dose to the larynx was 43.8 Gy (Gy) (± 14.5) and statistically significant across time the periods (*p* = 0.018, η2 = 0.161). No difference was found between age, HPV status, T stage, smoking history, or tumor site. The majority of head and neck cancer patients treated with chemoradiation who developed dysphagia did so within the acute time period (during treatment and up to 6 months post treatment). However, a substantial proportion of patients also developed dysphagia in later time periods (16%). The incidence of dysphagia in certain time periods may be impacted by laryngeal radiation dose. Therefore, we recommend long term monitoring/screening of these patients so early intervention can occur.

## Introduction

Head and neck cancer (HNC) encompasses patients with squamous cell carcinoma (SCC) of the upper aerodigestive tract mucosa and rare cancers occurring in the same anatomic location (e.g., adenocarcinoma, adenoid cystic carcinoma, and sarcomas) [1]. From 1975 to 2016 the incidence of HNC has increased on average 0.6% per year [2]. Despite this, the survival rate has steadily improved [2, 3], likely due to the favorable prognosis in younger patients with HPV associated oropharyngeal (OP) SCC [4–6], the radiosensitivity of HPV positive SCC [6], and advances in treatment modalities [1, 7, 8]. As the prognosis of HNC improves, the complications of treatment, like radiation-induced dysphagia (RID), are becoming more apparent [4, 9]. 

While chemoradiotherapy (CRT) is organ sparing, this does not imply intact function [10]. Unfortunately, even modern techniques like intensity modulated radiation therapy (IMRT) and TomoTherapy do not completely spare normal surrounding tissue, like the pharyngeal constrictor musculature, [11–12] cranial nerves [13], and cervical lymphatic drainage beds due their proximity and necessary inclusion in the radiation field. Radiotherapy causes cellular damage leading to an unregulated and imbalanced inflammatory reaction. Following the initial insult, tissue remodeling is driven by increased fibrocytes secreting transforming-growth factor – beta (TGF-B) resulting in increased collagen deposition and imperfect repair [11–14]. Late onset radiation-induced dysphagia (LORID) is a known complication of head and neck radiotherapy. The pathophysiology is not fully understood but likely secondary to fibrosis, lower cranial neuropathies, impaired sensation, microvascular insult, and/or pharyngeal constrictor atrophy [11, 14–18]. Decline of swallowing function has high morbidity as dysphagia leads to decreased quality of life, aspiration risk, weight loss, and social isolation. [1, 19, 20–21] Long term dysphagia has been shown to affect up to 59% of HNC patients following radiotherapy [22]. Despite this significant impact, a systematic review of swallowing outcomes in oropharyngeal HNC patients found that only 10% of the literature reported 5-year swallowing outcomes [23]. The lapse in long term outcomes could be due to the varied definitions of LORID in the literature spanning from > 90 days to > 5 years post radiation. [21, 23–24]

The risk factors and timeline for developing LORID post treatment are not fully characterized [25–28]. We aimed to investigate the development of dysphagia after definitive radiation treatment in HNC patients over a ten-year period at a tertiary referral center. We hypothesized that the development of RID varies over time with a subset of patients developing clinically relevant dysphagia in the late onset period.

## Materials and Methods

This study was approved by the Institutional Review Board (IRB Protocol 00119744). This retrospective study was conducted in accordance with the ethical standards set in the 1964 Declaration of Helsinki and its later amendments. As a retrospective study utilizing anonymized patient data from medical records, informed consent was waived by the IRB. The data that support the findings of this study are restricted based on the conditions of the IRB and are not publicly available.

**Fig. 1:**
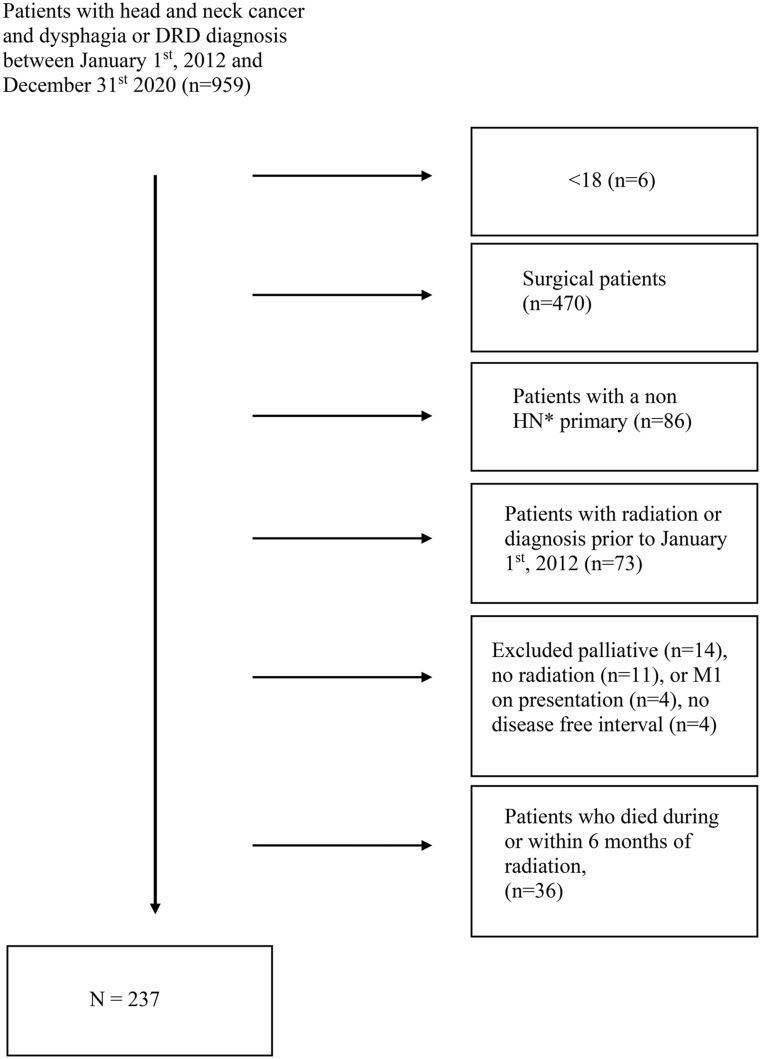
Exclusion criteria. Abbreviations HN, head and neck. Diagram displaying exclusion criteria

Adult patients (≥ 18 years) diagnosed with a head and neck malignancy and dysphagia or a dysphagia related diagnosis between January 1, 2012 and January 1, 2022 were included. Patients who underwent initial or salvage primary surgical resection, palliative radiation, prior head and neck radiation, presented with distant metastases or died within 6 months of treatment were excluded (Fig. 1). Those patients who underwent diagnostic tonsillectomy, operative biopsy, lymph node excision, and/or salvage neck dissections were included. Data collected from the patient’s electronic medical record also included demographics, cancer diagnosis, location, type, and treatment, and the dates of procedures.

To identify patients with dysphagia and dysphagia related diagnoses (DRD), International Classification of Diseases, Ninth Revision (ICD-9), International Classification of Diseases, 10th Revision (ICD-10) and Current Procedural Terminology (CPT) codes were used. The specific diagnoses included: unspecified dysphagia (ICD9: 787.2, 787.29 ICD10: R13.10, R13.19), oropharyngeal (ICD9: 787.22; ICD10: R13.12), oral (ICD9: 787.21; ICD10: R13.11), and pharyngeal/ pharyngoesophageal phase dysphagia (ICD9: 787.23, 787.24; ICD10: R13.13, R13.14). DRD included feeding difficulties (ICD9: 783.3; ICD10: R63.3), esophageal stenosis, stricture, and/or obstruction (ICD9: 530.3; ICD10:K22.2), malnutrition (ICD9: 263, 263.1, 263.8, 263.9); ICD10: E46), esophageal dyskinesis (ICD9: 530.5; ICD10: K22.4), dehydration (ICD9: 276.51; ICD10: E86.0), and abnormal weight loss (ICD9: 783.21; ICD10: R63.4). Dysphagia related procedures (DRP) were open, percutaneous, or laparoscopic gastrostomy tube placement (ICD9: 43.0, 43.11, 43.19; CPT: 43830, 43653, 43246,), modified barium swallow study (MBSS) (CPT 70370, 70371, 74230), flexible endoscopic evaluation of swallowing (FEES) (CPT 92610, 92612, 92616, 92611), and/or encounter for enteral nutrition (ICD9: 96.6).

Dysphagia patients were grouped into four time intervals: (1) 0–6 months, (2) 6 months − 1 year, (3) 1–2 years, and (4) greater than 2 years from radiation completion. To account for acute toxicities of treatment and baseline diagnostics, we excluded diagnoses before and during radiation. Once the diagnosis of dysphagia was made, patients were excluded from subsequent time intervals, even if dysphagia persisted, so that only new diagnoses were counted.

Radiation doses to organs at risk (OARs) were collected by the contours outlined by the treating radiation oncologist from the original radiation treatment plan. Presumed scatter dose to OARs was recorded as 0. All statistical analyses were performed using SPSS version 27.0.1.0 (IBM Corporation, Armonk, NY, USA). Categorical variables (T stage, age, HPV status, dysphagia diagnosis, etc.) were summarized by frequency (n) and percentage (%). Comparison of variables was performed using a Chi-square or Anova with Yates correction or Fisher’s exact test and effect sizes were calculated with Phi, Cramer’s V, or Eta squared as appropriate. Significance was set at *p* < 0.05.

## Results

“A total of 959 patients were screened for the study with 237 patients meeting criteria of head and neck cancer treated with radiation therapy and a diagnosis of dysphagia at any time point. (Fig. 1). Of these 237 patients, 71 (30.0%) were diagnosed with dysphagia prior to the end of radiation and 166 (70.0%) after completion of radiation. Patient demographics are shown in Table 1. The average age was 62 ± 12.6 years with 153 patients (64.6%) being less than 65 years. The cohort was predominantly male (*n* = 198, 83.5%) and white (*n* = 188; 79.3%). There were 165 patients (69.6%) with current or a history of tobacco use.

**Table 1:** Patient demographics and baseline characteristics

	*n* = 237
Age MN +/-SD	62 +/-12.6
< 65 n (%)	153 (64.6)
male	198 (83.5)
female	39 (16.5)
White	188 (79.3)
Nonwhite	49 (20.7)
H/o smoking	165 (69.6)
**HN subsite n (%)**	
Oral cavity	12 (5.1)
Nasopharynx + paranasal sinuses	15 (6.3)
Oropharynx	146 (60.8)
Hypopharynx	21 (8.9)
Larynx + laryngeal subsites	28 (11.8)
Unspecified larynx	3
Supraglottis	19
Transglottic	6
Subglottic	0
Unknown primary	12 (5.1)
Other (salivary gland, parapharyngeal space)	2 (1.0)
**Cancer type**	
SCC	232 (97.9)
HPV positive (n, % of SCC)	135 (58.2)
HPV negative	68 (29.3)
Unknown HPV status	29 (12.5)
Other (adenocarcinoma, angiosarcoma, neuroendocrine carcinoma, lymphoma)	5 (2.1)
**T stage n(%)**	
T0	3 (1.3)
T1	27 (11.4)
T2	61 (25.7)
T3	77 (32.5)
T4	57 (24.0)
Unknown (Tx)	12 (5.1)
**N Stage n (%)**	
N0	32 (13.5)
N1	55 (23.2)
1c	1 (0.4)
N2	49 (20.7)
N2a	4 (1.7)
N2b	36 (15.2)
N2c	42 (17.7)
N3	15 (6.3)
3b	1 (0.4)
Not listed	2 (0.8)
**Treatment**	
Tonsillectomy	21 (8.9)
Neck dissection	26 (11.0)
Radiation only	9 (3.8)
Adjuvant Chemotherapy + Radiation	13 (5.5)
Concurrent Chemotherapy + Radiation	209 (88.2)
Neoadjuvant Chemotherapy + concurrent chemoradiation	6 (2.5)
**Primary Chemotherapeutic agent**,** n (% of CRT patients)**	
Cisplatin	182 (79.8)
Immunotherapy (nivolumab or durvalumab)	5 (2.3)
Carboplatin/Paclitaxel	25 (11.0)
Paclitaxel	2 (1.0)
Cetuximab	11 (4.8)
Not listed	3 (1.3)
**Radiation Subsite Dose (Gy)**,** MN +/- SD**	
Right Parotid	29. +/- 11.2
Left Parotid	28.0 +/- 8.1
Larynx	43.8 +/- 14.5
Constrictors/ Pharynx	55.8 +/- 7.8
Hyoid	57.2 +/- 12.3
Oral Cavity	30.7 +/- 9.7
Time from diagnosis to radiation end (days, MN +/- SD)	105.04 +/- 44.4
**Neck Irradiation**,** n (%)**	
Bilateral Neck	211 (89.0)
Unilateral Neck	10 (4.2)
None	3 (1.3)
No Dosing Data	13 (5.5)
**Outcomes**,** n (%)**	
Recurrence	63 (26.6)
Alive as of 1/2022	186 (78.5)

Abbreviations MN, mean; SD, standard deviation; H/o, history of; HN, head and neck; SCC, squamous cell carcinoma; HPV, human papillomavirus; CRT, chemoradiotherapy

Most of the cohort were diagnosed with SCC (*n* = 232; 97.9%) of which 135 (58.2%) were HPV positive, 68 (29.3%) were HPV negative, and 29 (12.5%) had unknown HPV status. The five remaining patients were diagnosed with adenocarcinoma (*n* = 2), angiosarcoma (*n* = 1), neuroendocrine carcinoma (*n* = 1), or adenoid cystic carcinoma (*n* = 1).

The most common subsites were the oropharynx (*n* = 146, 60.8%), followed by the larynx (*n* = 28, 11.8%) and hypopharynx (*n* = 21; 8.9%). Advanced tumors (T3 and T4) and nodal stage (N2 and N3) constituted 131 (56.5%) and 147 (62.0%) of the cohort respectively. The individual Tumor, Nodal, and Metastatic (TNM) staging can be found in Table 1. All patients were treated with radiation (*n* = 9; 3.8%) or chemoradiation (total: *n* = 228; 95.8%) with chemotherapy being concurrent (*n* = 209; 88.2%), adjuvant (*n* = 13: 5.5%), or neoadjuvant (*n* = 6; 2.5%). Cisplatin was the predominate chemotherapeutic agent (*n* = 182; 79.8%). The remainder of CRT patients received primary carboplatin/paclitaxel (*n* = 25, 10.5%), paclitaxel (*n* = 2; 1%), targeted therapy (cetuximab (*n* = 11; 4.8%), or immunotherapy nivolumab (*n* = 3; 1.3%) durvalumab (*n* = 2; 1.0%). Three (1.3%) patients did not have the chemotherapeutic agent specified. The overall mean radiation mean dose to the right parotid, left parotid, larynx, constrictors/pharynx, and hyoid are outlined in Table 1. The majority of patients (*n* = 211; 89.0%) received bilateral neck irradiation. There were 13 (5.5%) patients who received radiation treatment at an outside institution. The mean time from cancer diagnosis to radiation end was 105.0 ± 44.4 days. A minority of patients required diagnostic tonsillectomy (*n* = 21, 8.9%) and/or diagnostic or salvage neck dissection (*n* = 26, 11.0%). At end of the study period, 186 (78.5%) of patients were alive and 63 (26.6%) had recurrence.

**Fig. 2:**
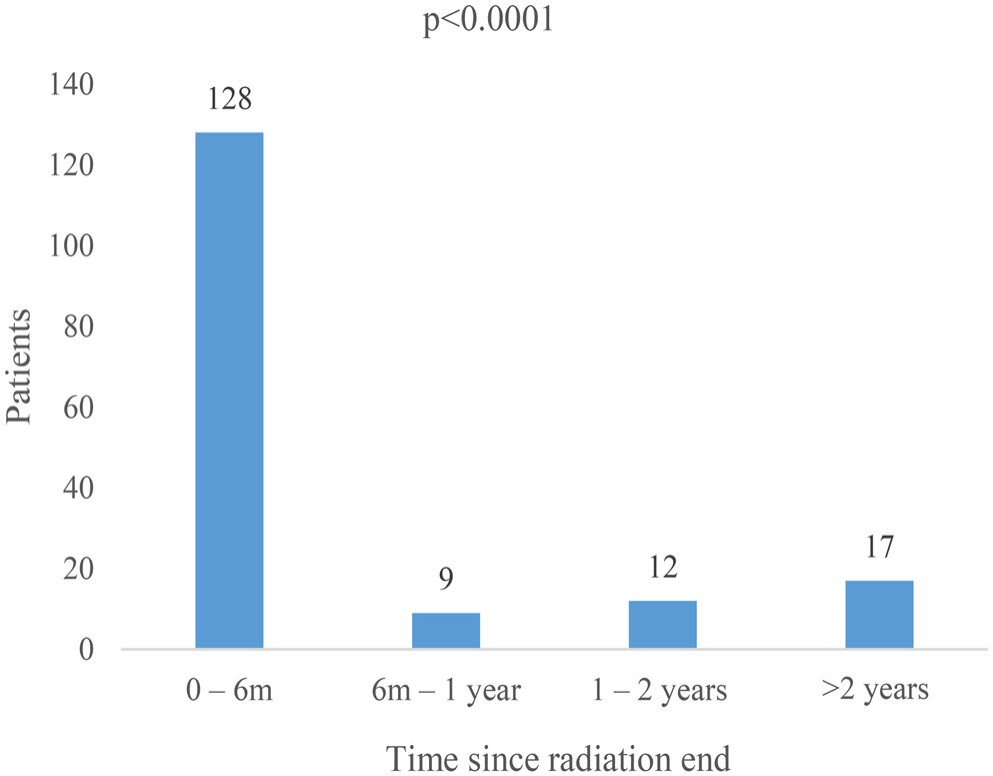
First incidence of any dysphagia diagnosis after treatment end. Abbreviations m, month. Bar graph demonstrating the time interval for the first dysphagia or dysphagia related diagnosis for each patient

The number of patients diagnosed with dysphagia or related diagnoses at each time period were 128 patients (54.0%) between 0 and 6 months, 9 (3.8%) between 6 months − 1 year, 12 (5.1%) between 1 and 2 years, and 17 (7.2%) at greater than 2 years from radiation end (*p* < 0.001) (Fig. 2; Table 2). There was no significant difference between dysphagia diagnosis across time intervals when patients with diagnoses in the acute period (0–6 months) were excluded (*p* = 0.28). Many patients had multiple diagnoses within each time interval as shown in Table 2.

**Table 2:** Dysphagia diagnoses (based on ICD-9/ICD-10 code) at each time interval

	0–6 m	6 m – 1 year	1–2 years	> 2 years	Total diagnoses*	*p*	Cramer’s V
First incidence of any dysphagia diagnosis, n (%)	128 (54.0)	9 (3.8)	12 (5.1)	17 (7.2)	166	< 0.0001	0.1
**Individual diagnoses**							
Dysphagia, Oral/ Oropharyngeal phase	48	8	9	15	80		
Dysphagia, Pharyngeal/ pharyngoesophageal phase	2	2	3	7	14		
Dysphagia, Unspecified	49	10	14	14	87		
Gastrostomy tube	14	5	4	7	30		
Enteral Nutrition	3	1	0	1	5		
MBSS	31	4	10	10	55		
Dehydration	61	3	3	7	74		
Feeding Difficulties	2	4	2	2	10		
Abnormal weight loss	47	3	5	10	65		
Malnutrition	15	0	2	3	20		
Esophageal Obstruction	4	3	2	4	13		
Esophageal Stenosis	5	2	0	0	7		

*Note that 71 patients were diagnosed with dysphagia prior to the end of radiation treatment (< 0 months) and are not included in this Table

For the diagnosis of dysphagia after radiation, there was no significant difference across time intervals for T stage (*p* = 0.475), age (*p* = 0.207), smoking history (*p* = 0.503), or HPV status for all sites (*p* = 0.103) or oropharyngeal cancer (*p* = 0.587) (Table 3). There was also no difference for cancer subsite (*p* = 0.144) (Table 4).

**Table 3:** First incidence of any dysphagia diagnosis

	0–6 m	6 m -1 year	1–2 years	> 2 years	*p*
**T stage**					0.475
T0	1	0	1	0	
T1	11	0	3	2	
T2	36	2	4	5	
T3	44	4	2	5	
T4	28	3	2	5	
Unknown (Tx)	8	0	0	0	
**Age**					0.207
< 65	82	8	8	8	
≥ 65	46	1	4	9	
**Smoking history**					0.503
Yes	84	7	8	14	
No	44	2	4	3	
**HPV status: all sites**					0.103
HPV +	78	4	8	7	
HPV -	35	5	0	7	
Unknown	13	0	2	3	
**HPV status: Oropharyngeal cancer**					0.587
HPV +	63	3	7	5	
HPV -	18	1	0	3	
Unknown	4	0	1	0	

Abbreviations m, months. Table displaying risk factors for dysphagia incidence at each time interval

**Table 4:** Cancer subsite and first incidence of dysphagia/ DRD or DRP

	0–6 months	6 m – 1year	1–2 years	> 2 years	*p*	Cramer’s V
					0.144	0.22
Oral Cavity	3	0	0	2		
Oropharynx	85	4	8	8		
Nasopharynx, nasal and paranasal sinus	8	0	2	1		
Hypopharynx	10	3	0	1		
Larynx	13	2	1	5		
Unknown Primary	7	0	1	0		
Other	2	0	0	0		
Total:	128	9	12	17		

The larynx, constrictors/pharynx, parotid glands, hyoid, and oral cavities’ mean radiation dose across time intervals of dysphagia incidence are displayed in Table 5. The larynx mean radiation dose was statistically different across time intervals (*p* = 0.018, η2 = 0.161). The mean larynx radiation dose across all time periods was 43.8 +/- 14.5 Gy whereas patients who presented at 6 months – 1 year with a new diagnosis received a greater radiation dose at 66.1 +/- 1.3 Gy. There was no significant difference for the diagnosis of dysphagia and mean radiation to the remaining OARs (all *p* > 0.05) (Table 5).

**Table 5:** Organ subsite radiation mean dose and first incidence of dysphagia/ DRD or DRP

	0–6 months	6 m – 1year	1–2 years	> 2 years	*p*	η2
Right Parotid MN +/- SD	29.5 +/- 11.0	32.9 +/- 16.0	31.4 +/-14.2	30.7 +/- 7.6	0.807	0.031
Left Parotid	28.2 +/- 7.4	27.0 +/- 8.1	23.0 +/- 12.5	30.4 +/- 9.2	0.156	0.920
Larynx	42.8 +/- 14.2	66.1 +/- 1.3	39.6 +/- 17.2	52.4 +/- 10.3	0.018	0.161
Constrictors/ Pharynx	56.1 +/- 7.2	52.4 +/- 8.1	50.8 +/-11.1	56.9 +/- 9.2	0.182	0.106
Hyoid	58.2 +/- 11.9	58.3 +/- 10.8	52.3 +/- 17.4	54.8 +/- 12.0	0.619	0.092
Oral Cavity	30.5 +/- 0.3	33.7+/- 13.1	31.2 +/- 9.4	30.2 +/- 11.6	0.056	0.030

## Discussion

The literature regarding radiation-induced fibrosis is limited to mixed treatment cohorts focusing on dysphagia severity or radiation dose and modality. As late radiation effects are known to worsen over time, [29–30] we aimed to identify the clinically relevant time interval and risk factors for radiation-induced dysphagia in nonsurgical patients. The pathophysiology of acute radiation-induced dysphagia is attributed to cells with high turnover (e.g., dermatitis or mucositis) developing within 90 days [31] and resolving within 6 months [32]. The definition of late onset radiation complications is not standardized. The definition of “late” or “very late” dysphagia ranges from > 90 days [31], 6 months, [22–23] 2 years [10, 17], 2.5 years [33], 3 years [29], to greater than 5 years [17, 24]. The pathophysiology is not fully understood but is likely secondary to imperfect tissue remodeling causing a combination of fibrosis, impaired blood flow, muscular atrophy and neuropathy after the acute effects of radiation resolve. Radiation-induced fibrosis peaks at 12 to 18 months after radiation; therefore, many studies may be missing a critical time interval as patients one year post treatment are still at risk for developing dysphagia [11, 14]. The present study evaluated smaller time intervals in order to identify clinically relevant trends and possible predictors for new dysphagia diagnoses.

In the literature, most studies evaluate dysphagia severity over time, inadvertently excluding patients with new diagnoses [10, 22, 29, 33, 34]. Few studies evaluate new dysphagia over time and are limited to mixed treatment modalities (surgery, chemotherapy, and/or radiation) [24]. In the present study, of patients with HNC that developed dysphagia, 5.1% and 7.2% presented at 1–2 years and greater than 2 years, respectively. This demonstrates that new late onset dysphagia is a lingering threat for patients despite intervals of adequate function. Importantly, these patients are distinct from those with progressive dysphagia persisting months to years following treatment.

Radiation dose and its correlation to swallowing impairment has been demonstrated [34–39]. Of the 25 muscles and 5 cranial nerves required for deglutition, the predictive radiation dose for muscle and nerve dysfunction varies in the literature. [13, 33–34, 39] However, overall pharyngeal constrictor muscle dose has consistently been identified as a predictor for dysphagia. [40–41] In our study, the mean radiation dose to the constrictor muscles and hyoid were higher than the other OARs stratified, but not significantly different across time intervals. This is also consistent with previous literature. Mogadas et al. [39] found that, after 24 months, radiation dose to the pharyngeal constrictor muscles no longer predicted dysphagia in HPV positive oropharyngeal cancer patients. Ray et al. [42] found no relationship between radiation dose to pharyngeal constrictors and cricopharyngeal muscles and late dysphagia score at > 6 months.

The mean radiation dose to OARs as outlined by the treating radiation oncologist demonstrated that radiation to the larynx significantly impacted the incidence of dysphagia. Patients who received greater radiation dose to the larynx experienced new dysphagia diagnoses at 6 months – 1 year and > 2 years. An overwhelming majority of our patients received unilateral or bilateral cervical irradiation as 86% (*n* = 203) had nodal disease in initial staging. The cervical lymphatic beds’ proximity to the neuromuscular structures involved in the complex coordination of swallowing and the linear relationship between time and neck fibrosis places these patients at higher risk for dysphagia [29, 36]. 

There are mixed results in the literature regarding risk factors as predictors of late dysphagia [22, 29, 35]. For example, Meyer et al. [35] found female gender and weight loss during radiation were independent predictors of toxicity at 12 months. Toxicity was measured by the first version of the Radiation Therapy Oncology Group (RTOG) Acute Radiation Morbidity Scoring which included severe/ critical weight loss as a criterion for grade 3 toxicity for the pharynx and esophagus [31]. Therefore, patients with feeding difficulties during radiation could be at higher risk for later dysphagia and/or feeding difficulties [31, 35]. In contrast, Szczesniak et al. [22] found patient age, gender, time since therapy, tumor site, and tumor stage did not predict dysphagia severity. Baudelet et al. [29] found patients with advanced tumors (T0-2 vs. T3-4) and older age had more severe dysphagia. Ray et al. [42] found only oropharyngeal cancer and HPV status as predictive of late dysphagia. Additionally, acute toxicities prognostic value in predicting late dysphagia is debated [12, 43]. The present study found no difference in dysphagia incidence based on age, tumor site, HPV status, smoking history, or T classification.

Chemotherapy is known to exacerbate acute radiation associated toxicities [8, 10, 32, 44] and notably, 228 (96.2%) of our patients received chemotherapy. The non-chemoradiotherapy group was limited and therefore we were unable to analyze differences between these populations. The predictive factors for LORID are more likely to be a complex interplay of tumor burden, treatment, and inherent genetic differences in reaction to radiation and chemoradiotherapy. [17, 21–22, 28]

We found that 12.7% (30/237) patients had a gastrostomy tube placed after the end of treatment. Unfortunately, the study design does not provide insight into the context of gastrostomy tube placement. However, it is reasonable to assume but not proven by this dataset that the onset of dysphagia following treatment could be a contributing factor as prophylactic gastrostomy tube placement is generally avoided at our institution. Anecdotally, in our practice, we have observed that patients may require gastrostomy tube placement well after their treatment has concluded. The proportion of nonsurgical patients requiring a gastrostomy tube in the months to years following radiation treatment highlights the morbidity of definitive radiation therapy and impact on quality of life [1, 19, 20, 41]. 

### Limitations

It is possible our retrospective methodology did not include all patients with late dysphagia. Szczesniak et al. [22] found a discordance in patient’s subjective swallowing dysfunction and electronic medical record (EMR) documentation as the afferent nerve damage could minimize a patient’s subjective sensation and lead to underreporting [13, 22]. Our study was limited to the presence of diagnostic codes in the electronic medical record. Therefore, the context in which certain procedures took place, such as gastrostomy tube placement, is lacking. Additionally, we did not extract data from diagnostic procedures such as videofluoroscopy or FEES and so were unable to characterize the severity of dysphagia. Lastly, although speech-language pathologists (SLP) are consistently involved in head and neck cancer care at our institution, including participation in preoperative clinics and Tumor Board, we could not reliably quantify that all patients received evaluation by SLP prior to radiation. It is important to note that our institution does not routinely empirically perform instrumental examinations or prophylactic gastrostomy on patients undergoing radiation therapy; instead, these interventions are guided by clinical indications. Future studies should aim to evaluate the extent, progression, and/or recovery of radiation-induced dysphagia over time.

The data presented are inherently limited to the electronic medical record and physician coding practices. Patients were enrolled in various radiation protocols by different physicians which limit the comparison across OAR dosing. Additionally, the TNM staging was recorded from the initial radiation oncologists’ notes. Our data included patients between 2012 and 2022, therefore including both AJCC 7th and 8th TNM classifications.

## Conclusion

The majority of head and neck cancer patients treated with chemoradiation who developed dysphagia did so within the acute time period (during treatment and up to 6 months post-treatment). However, a substantial proportion of patients also developed dysphagia in later time periods (16.0%). Therefore, we recommend long term monitoring/screening of these patients so early intervention can occur.

## Data Availability

The data that support the findings of this study are restricted based on the conditions of the MUSC IRB and are not publicly available.
